# Three-Dimensional Accuracy of Digital Impression versus Conventional Method: Effect of Implant Angulation and Connection Type

**DOI:** 10.1155/2018/3761750

**Published:** 2018-06-04

**Authors:** Marzieh Alikhasi, Hakime Siadat, Alireza Nasirpour, Mahya Hasanzade

**Affiliations:** ^1^Dental Research Center, Dentistry Research Institute, Department of Prosthodontics, Tehran University of Medical Sciences, Tehran, Iran; ^2^Dental Student Research Center, School of Dentistry, Tehran University of Medical Sciences, Tehran, Iran; ^3^Dental Research Center, Dentistry Research Institute, Department of Prosthodontics, School of Dentistry, Tehran University of Medical Sciences, Tehran, Iran

## Abstract

**Purpose:**

The aim of this in vitro study was to compare the accuracy of different implant impression techniques of the maxillary full arch with tilted implants of two connection types.

**Materials and Methods:**

Two maxillary edentulous acrylic resin models with two different implant connections (internal or external) served as a reference model. Each model had two anterior straight and two posterior angulated implants. Ninety impressions were made using an intraoral scanner (Trios 3Shape) with scan bodies for digital impression (groups DII and DIE), a custom open tray with additional silicone for the conventional direct group (groups CDI and CDE), and a custom closed tray with additional silicone for the conventional indirect group (groups CII and CIE) from both internal and external models, respectively. A coordinate-measuring machine (CMM) was used to measure linear and angular displacement for conventional specimens. For digital groups, an optical CMM was used to scan the reference model. STL data sets from the digital specimen were superimposed on STL reference data sets to assess angular and linear deviations. Data were analyzed with three-way ANOVA and *t*-test at *α*=0.05.

**Results:**

There were significant angular and linear distortion differences among three impression groups (*P* < 0.001), angular distortion differences between internal and external connections (*P* < 0.001), and between straight and tilted implants for either linear (*P* < 0.001) or angular (*P*=0.002) distortion. The type of the connection and implant angle did not have any effect on linear and angular distortion of the digital technique (*p* > 0.05). Minimum angular and linear distortion was seen for tilted implants in DII and DIE groups (0.36° ± 0.37 and 0.16 ± 0.1 mm).

**Conclusion:**

Impression techniques (digital versus conventional) affected the transfer accuracy. Digital techniques demonstrated superior outcome in comparison with conventional methods, and the direct technique was better than the indirect conventional technique. Connection type and implant angulation were other factors that influenced accuracy. However, when digital impression was applied, accuracy was not affected by the type of connection and angulation.

## 1. Introduction

High precision in transfer of clinical conditions to dental laboratory is one of the most important factors in fabrication of the prosthesis with excellent fit for either natural teeth or implants [1]. Therefore, the essential first step for fabrication of a successful implant-supported prosthesis is accurate transfer of three-dimensional implant position and angulation from the mouth to the master cast via impression [1, 2]. Inaccurate position of the implant in the master cast makes it impossible to fabricate a well-fitting prosthesis, and the resultant misfit can lead to biomechanical complications such as screw loosening [3], bone loss [4], and ceramic veneer fracture as a result of increasing stress within the prosthesis or at the interface of the implant and bone. Accuracy of the master cast is influenced by several factors including the impression technique, type of the tray [5, 6], manipulation of the dental stone, and its compatibility with the impression material [7]. Each step could have a potential error related to inherent materials or humans which is inevitable. Moreover, other factors involved in the precision of implant impression could be the impression technique (direct versus indirect), splinting, machining tolerance of components, number and angle of implants, depth of implants, and type of connection [8–14]. Multiple implants with different angulations can cause distortion of the impression material on removal [11]. In a review by Lee et al. [15], it has been reported that when the implants are more than three, angulation of implants may affect the accuracy. However, when the implants are limited to 2 or 3, no effect was reported on the impression accuracy [10]. Also, many articles studied the accuracy of different implant impression techniques [16–20]. For situations in which there were 4 or more implants, studies showed more accurate impressions with the direct technique than the transfer technique [8, 15, 18, 19].

The advent of intraoral scanners (IOSs) has led to a change in implant dentistry. Although the first IOSs became commercially available two decades ago, their popularity in recent years has grown dramatically, which results from an increase in precision and efficiency [21]. Digital impression can improve patient acceptance [22], reduce possible distortion of impression materials and master casts [11], reduce chairside time [22], and provide a 3D image of preparation. Although some articles reported distortion and lower accuracy for digital impression [5, 17], there is also some defensive evidence that shows digital impression comparable to or even better than conventional impression [16, 21, 23, 24]. Therefore, there is disagreement towards the priority of these methods.

Fabrication of the prosthesis with CAD/CAM has many steps including acquisition of data by scanning, processing the information, designing the restoration, and eventually manufacturing. All of these steps have some potential errors which are displayed in the final restoration as the amount of misfit. As different factors influence the accuracy of each step, breaking up of the errors of different steps is important. Several studies have compared fitness of final restoration fabricated with CAD/CAM or conventional methods [12, 25–28] although there is not enough knowledge of the accuracy of intraoral digital impression systems for dental implants [11, 29].

The aim of the current study was to compare the accuracy of conventional (direct and indirect) and intraoral digital impressions of the maxillary full arch with tilted implants of two connection types. The null hypothesis was that there was no difference between digital and conventional techniques, and also, implant angulation and connection type would not affect the accuracy.

## 2. Materials and Methods

This in vitro experimental study was conducted on two edentulous maxillary acrylic resin models with two different implant connections (internal trilobe and external hexagon). In each resin model, the two anterior implants were placed straight at the site of canine teeth with no angulation and parallel to each other (their longitudinal axis was perpendicular to the plane of the resin model). Implants were numbered from 1 to 4 from the posterior right to the posterior left (implants 1 and 4 were angulated and 2 and 3 had straight position). The two posterior implants were placed at the site of the second premolars with 45° distal angulations. The NobelReplace implant system (Nobel Biocare AB, Göteborg, Sweden) was used in one acrylic resin model with a regular diameter (4.3 mm), 11 mm height, and internal trilobe connection. The Branemark Nobel Biocare implant system (Brånemark System® Mk III, Nobel Biocare AB) with a regular diameter (4.1 mm), 12 mm height, and external hexagon connection was used in the second acrylic resin model. A metal reference cylinder was inserted in the midline of the palate in the model as a reference of measurement and was defined as the zero point [13].

Description of the groups is presented in Table 1. After 24 hours, the conical impression copings of both systems (Nobel Biocare AB, Göteborg, Sweden) were fastened to the implants, the baseplate wax (Modeling wax; Dentsply DeTrey, Konstanz, Germany) was adapted around and over the impression coping, and irreversible hydrocolloid (Alginoplast; Heraeus Kulzer GmbH &Co., Wehrheim, Germany) impressions were made to obtain two casts. These casts were used to mold custom trays. The obtained casts were covered by two layers of the baseplate wax (Modeling wax; Dentsply DeTrey, Konstanz, Germany) to allow a reliable thickness of the impression material. Tissue stops were included in the impression trays to standardize tray positioning during impression making. Sixty 2 mm thick custom impression trays (30 open trays and 30 closed trays) were made with light polymerizing resin (Megatray; Megadenta, Radeberg, Germany). Each tray was perforated, and the internal part and 5 mm outside of the borders were coated with an adhesive 30 minutes before each impression was made. Addition silicone (Zhermack Elite HD + Regular Body, Kouigo, Italy) was the impression material of choice for all transfer procedures and was managed according to manufacturers' recommendations and the specification number 19 of ADA. All impressions were made in a temperature-controlled environment (23 ± 1°C) with a relative humidity of 50 ± 10% [13].

Square copings in groups CDI and CDE and conical copings in groups CII and CIE were adapted to the implants. All impression copings were secured with a torque wrench calibrated at 10 Ncm torque on the implants. An automixing cartridge was used for mixing the impression material. For each impression, 12 mL of the material was carefully injected around and over the copings to ensure complete coverage of the copings. 35 mL of the remaining impression material was used to fill the impression special tray. To standardize the seating load for each impression, a 5 kg weight was placed over the trays during material polymerization. The impression materials were allowed to polymerize for 12 minutes after the start of the procedure according to the manufacturer's recommendation. The impression/matrix set was placed in distilled water at 36 ± 1°C during the setting time.

Once the impression had been obtained, implant analogues were adapted and screwed into the pick-up impression copings. In groups CII and CIE, the impression/matrix set was separated. Then, the conical transfer impression copings were unscrewed from the matrix and fitted to the implant analogues, and they were immediately replaced in each respective notch left in the impression. The combined impression coping analogue unit was inserted into the impression by firmly pushing it into place to full depth and slightly rotating it clockwise to feel the antirotational resistance. Casts were made by pouring type IV dental stone (Herostonel Vigodent Inc., Rio de Janeiro, RJ, Brazil), which was vacuum mixed with a powder/water ratio of 30 g/7 mL, as recommended by the manufacturer's instructions. When set (120 minutes after pouring), the impression was separated from the cast. The same operators prepared all sixty impressions in all clinical and laboratory procedures [13].

For making digital impression, Trios 3Shape (3Shape, Copenhagen, Denmark) IOS was used. Scan bodies for internal (14.005; DESS Abutments Co., Barcelona, Spain) and external (14.002; DESS Abutments Co., Barcelona, Spain) connections were torqued 10 Ncm to the NobelReplace and Branemark Nobel Biocare implants, respectively. Fifteen scans of the models were done by one experienced operator of each model. After calibrating and scanning by the operator, the best method selected was starting scan from the reference pin in the palate of the model towards the right tuberosity and lingual surfaces of all scan bodies. Next, the buccal surfaces and then the occlusal surfaces of scan bodies were scanned. Care was taken to well record the connection area and smooth surface at the distance between scan bodies. Intraoral scanning data were transferred to Dental System software and converted from 3OXZ format to STL format.

### 2.1. Measurements

**Figure 1:**
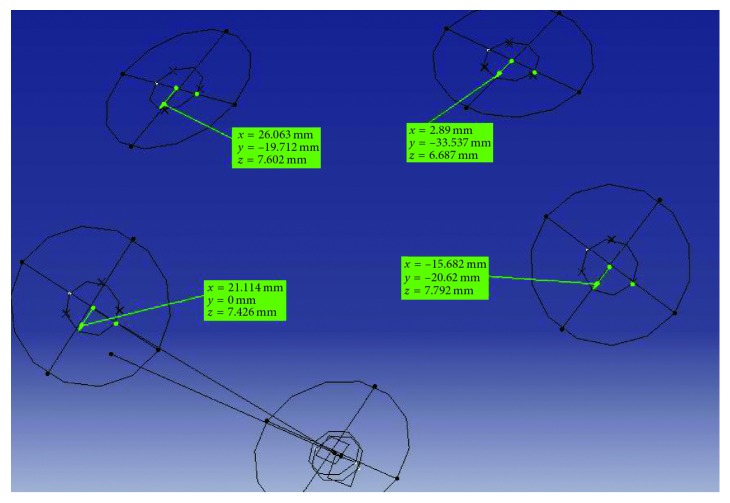
Schematic drawing of the measurements according to the reference point.

A single calibrated blinded examiner performed all readings randomly without any notification of previously described information about the code of each stone cast. The coordinate-measuring machine (CMM) (Mistral, DEA Brown&Sharpe, Grugliasco, Italy) was used for recording the *x-*, *y-*, and *z-*dimensions and also angular dislocation simultaneously. Each working cast was measured three times, and an average was obtained. Additionally, readings were obtained in each of four implants of the groups. These linear and angular measurements performed on the master models were repeated for all study casts. To represent three-dimensional linear displacement, Δ*r* was calculated using Δ*r*^2^=Δ*x*^2^+Δ*y*^2^+Δ*z*^2^, where Δ*x*, Δ*y*, and Δ*z* were displacements at *x*-, *y*-, and *z*-directions, respectively (Figure 1) [13].

**Figure 2:**
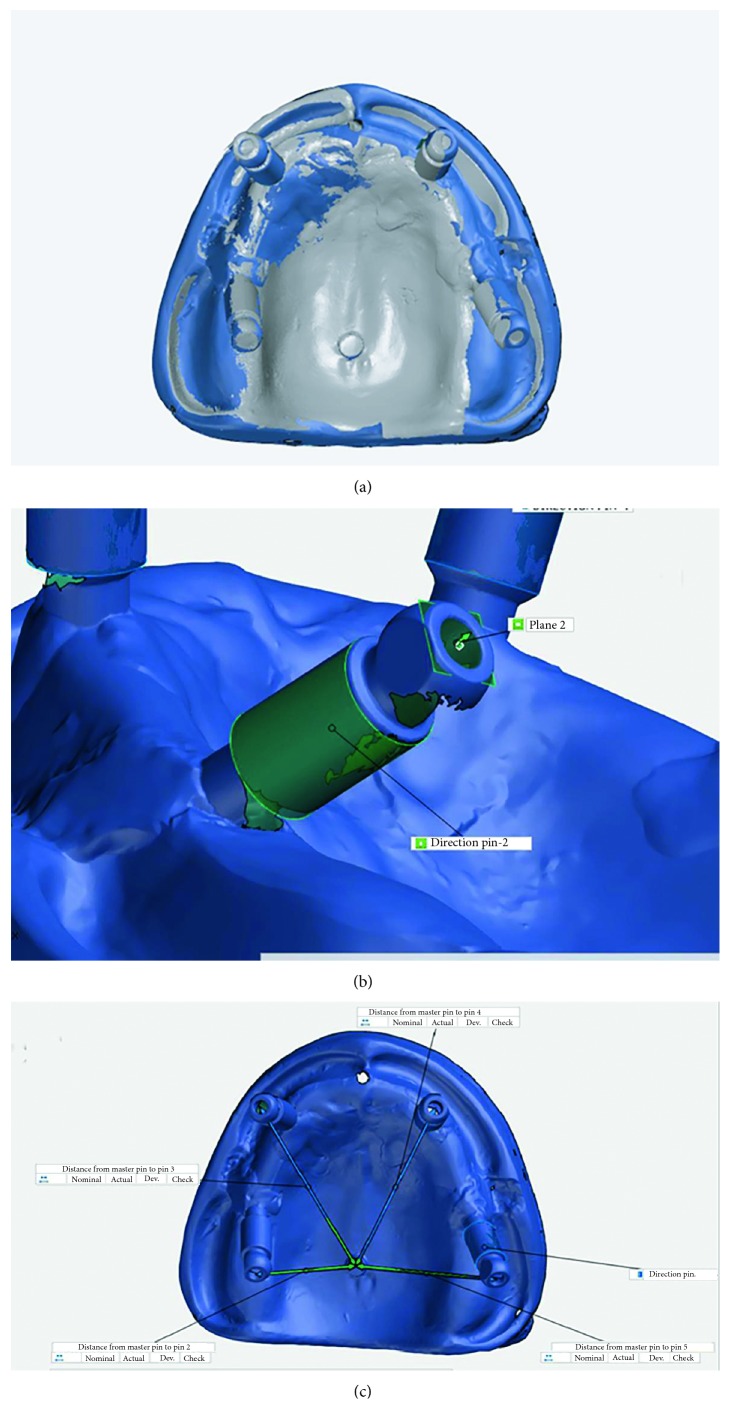
Digital impression measurements. (a) Superimposition of nominal and actual data. (b) Fitting plane and cylinder and intersecting point (green) which indicates implant position. (c) Linear measurements.

For digital models, reference models were scanned by an optical coordinate-measuring machine (ATOS Core 80; GOM GmbH, Germany). The data from this scanner were transferred to GOM Inspect software (GOM GmbH, Germany) in STL format and were set as a nominal element. The output data in STL format of intraoral scans were also transferred to GOM Inspect software as actual elements, and comparison with nominal values was made. Measurements were made by one experienced operator. For the measurements, first, the best-fitted plane to the occlusal surface of the reference pin and the scan bodies in each reference model were defined in the software, and then, a cylinder with the best fit to the external surface of each scan body was designed. The central axis of each cylinder was determined, and its intersection with the occlusal plane was marked (Figure 2(a)). The same definitions for the plane, cylinder, axis of each cylinder, and its intersection with the occlusal plane were used in the scanned model by Trios 3Shape and also for the reference pin in the palate. Then, the best-fit alignment was used for superimposition of scans obtained from Trios 3Shape on the corresponding images obtained from ATOS Core (Figure 2(b)). To determine the change in implant position, the distance between the intersection point of the central axis of the cylinder with the occlusal plane on the surface of the scan body and the central index was recorded. The reported deviation was the distance of the measured data point from the surface of the nominal (CAD) model to the actual model surface at that point location (Figure 2(c)). The software measured the values in three spatial planes of *XY*, *XZ*, and *YZ*. To determine the change in angular position of each scan body, the change in the angle of the cylinder axis of each scan body with the corresponding axis on the nominal model was calculated in degrees. All measurements were made automatically by the software.

### 2.2. Statistical Analysis

The sample size was calculated for 80% power, using PASS Sample Size Software version 11. Data were analyzed using SPSS version 23 (SPSS Inc., IL, USA). The mean and standard deviation values were reported for dependent variables including Δ*R* and Δ*A*. Considering the presence of three independent variables (impression method, implant connection, and implant angulation), three-way ANOVA was applied. Since the interaction effect of some independent variables was found to be significant, pairwise comparisons were done with post hoc Tukey and independent *t*-tests. The level of significance was set at 0.05.

## 3. Results

The mean and SD of the linear and angular distortion of six groups and subgroups are presented in Table 2. Three-way ANOVA showed a statistically significant difference among three impression techniques (*P* < 0.001), between internal and external connections regarding angular distortion (*P* < 0.001), between straight and tilted implants for either linear (*P* < 0.001) or angular (*P*=0.002) distortion, and their mutual interaction (*P* < 0.001). Minimum angular and linear distortion was seen for tilted implants in DII and DIE groups, respectively (0.36° ± 0.37 and 0.16 ± 0.1 mm). The maximum value of angular distortion was for tilted implants in the CII group (9.37° ± 6.9 mm), and straight implants in the CII group had maximum linear distortion (0.88 ± 0.38 mm).

### 3.1. Impression Method

The effect of the impression technique by comparing inaccuracy values for each group at the implant angulation and connection type is shown in Table 3. There was a significant difference of angular distortion (Δ*A*) among three impression groups (*P* < 0.001); The DII group produced better results than conventional direct and indirect techniques with either straight (*P* < 0.001) or tilted (*P* < 0.001) implants. The DIE group was more accurate than the CIE group both for straight (*P* < 0.001) and tilted (*P* < 0.001) implants. The DIE group showed more accurate values than the CDE group only for tilted implants (*P* < 0.001). Comparing direct and indirect methods, results showed that the direct technique (CDI and CDE groups) was more accurate than the indirect method (CII (*P* < 0.001) and CIE (*P* < 0.001)).

Linear distortion (Δ*r*), when external connection was used regardless of being straight or tilted digital technique, was better than both conventional direct (*P* < 0.001) and indirect (*P* < 0.001) methods. However, when the connection was internal, the DII group was more accurate than the CII group (*P* < 0.001) and the CDI group was better than the CII group (*P* < 0.001). There was no significant difference between digital (DII) and direct (CDI) techniques in straight implants.

### 3.2. Connection Type

The effect of the connection type was analyzed by comparing angular and linear distortion for each group with the impression technique and angulation of the implant as variables, and the results are demonstrated in Table 4. The results showed that there was no significant difference between internal and external connections of digital groups (DII and DIE). In conventional direct groups (CDI and CDE), the external connection was better than the internal connection in angular distortion both for tilted (*P* < 0.001) and straight (*P* < 0.001) implants. With the indirect impression technique (CII and CIE groups), the connection type did not have any effect on the accuracy of straight implant transfer, although for tilted implants, external connections showed better results of Δ*A* (*P* < 0.001) and Δ*r* (*P*=0.001).

### 3.3. Implant Angle

The effect of the implant angle was analyzed in the same way, and the results are shown in Table 5. There was no significant difference between angled and straight implants for the digital technique (DII and DIE groups). In the CDI group, straight implants were better than tilted implants regarding ∆A (*P* < 0.001) and Δ*r* (*P*=0.03). However, in the CIE group, tilted implants showed less Δ*r* (*P* < 0.001) and Δ*A* (*P* < 0.001) distortion.

## 4. Discussion

A precise impression of implants in an edentulous jaw is a prerequisite of an accurate master cast which is necessary for fabricating a well-fitting prosthesis [2]. The use of IOS is overgrowing; however, there is not enough evidence about the accuracy of it in comparison with the conventional method [8, 30]. The current study compares both the linear and angular distortion among three different impression methods (digital impression with 3Shape IOS, the conventional direct impression technique, and the conventional indirect impression technique), types of connections, and angulations of implants.

The null hypothesis was rejected as results demonstrated that digital impression has significantly less angular and linear distortion than conventional methods. However, digital impression of straight implants with internal connection was more accurate than that of the direct technique although the difference was not significant. These results verified findings of other studies which show that the impression technique could affect the transfer accuracy [16, 23]. A study by Amin et al. compared the accuracy of digital implant impressions using CEREC Omnicam and 3M True Definition versus conventional impression techniques for a five-implant full-arch edentulous mandible [16]. The authors reported that digital implant impressions were more accurate than conventional direct splinted implant-level impressions. Another in vitro study by Papaspyridakos et al. compared the accuracy of digital implant impression using the 3Shape scanner with the conventional method and showed that the accuracy of digital impression was comparable to that of the conventional method [23]. Digital impressions of five mandibular implants resulted in similar accuracy to the splinted implant-level impressions, and both techniques were superior to the nonsplinted, implant-level impression technique [23]. The result of this study is somehow in contrast with our study that can be related to factors such as the impression material, impression technique, expansion of stone, pouring stone technique, and machine tolerance of the prosthetic component. Moreover, different scan bodies of two studies could be an additional factor for contrary results.

Differences in the method for accuracy measurement is another contributing factor. In this study, the CMM was used for scanning casts from conventional impression to use that data set file as the comparative value with data from digital impression. However, Papaspyridakos et al. [23] scanned all stone casts with a 6 *µ* precision scanner (IScan D103i; Imetric), and data in the STL format were used to be compared with data from digital impression. Moreover, one of the implants was used as a reference for superimposition of the scans while using an unstable reference for superimposition is not a reliable technique.

Other factors that could influence the accuracy of impressions are implant angulation and connection type although evidence is insufficient in this field [8]. The results of the current study showed that when angulation was increased up to 45 degrees, accuracy was not affected in digital (DII and DIE) groups. Logically, accuracy of digital impression should not be affected by the angulation of implants as the concern of impression material deformation during removal, or displacement of impression coping is not an issue in this technique. In the conventional direct group, results vary depending on the connection type; In the CDI group, straight implants were better than tilted implants, but surprisingly, in the CDE group, tilted implants had less linear distortion than straight implants. Also, in the CIE group, tilted implants showed better accuracy than straight implants which can be explained by the fact that, in conventional impressions, the operator may remove the tray unexpectedly in direction of the tilted implant to prevent distortion.

In contrast to our result, Lin et al. reported that the divergence between the two implants (0, 15, 30, and 45 degrees) did not affect the accuracy of the definitive cast created through traditional impression, but the divergence between the two implants significantly affected the accuracy of the milled cast through digital impression [31]. They found that, at lower levels of divergence (0 to 15 degrees), conventional impression was more accurate than digital impressions. However, at a higher divergence (30 to 45 degrees), the differences in accuracy between conventional and digital impressions became less noticeable, with conventional impression still being slightly more accurate. The source of these contradictory results may be using different scanners and software (Cadent iTero) and different scan bodies (Straumann). Moreover, in the Lin et al. [31] study, milled polyurethane casts were fabricated from digital data, and implant analogues were inserted manually which can be a source of error. Chia et al. reported that, in the presence of angulated implants, there is a little difference between the digital impression and conventional technique [10]. Direct comparison between the results of our study and Chia et al. [10] should be done with caution, as the model in that study was partially edentulous and was restricted to two implants and the most angled specimen had 20-degree angulation although in our study, it was 45 degrees. A clinical study showed that digital impression for the all-on-four system with two straight and two tilted implants resulted in accurate physical models and improved efficiencies for the dental team [32].

The type of connection can affect the stability of the implant-prosthesis interface [12]. The Branemark system was characterized by external hexagon connection; this configuration has some weakness because of limited height; it is not efficient when the off-axis load is applied to resist micromovements [33]. However, this configuration may become a privilege during impression as it allows easier removal of the tray. In internal connection, the impression coping has an intimate fit within the implant which may make removal of the impression more difficult and may generate a higher degree of distortion. Based on the results of the current study in the conventional impression group, external connection implants showed less distortion than the internal one. As in digital impression, removing the impression is not an issue, and the type of connection does not influence the accuracy. In confirmation with our result, Papaspyridakos et al. [12, 23] reported that the type of connection influenced the accuracy at implant-level impression.

Different IOSs were used in studies, and it has been shown that the accuracy of scanners differs from each other, either for tooth or implant impression [26, 34]. In a study by Vandeweghe et al. [35], four different IOSs were used to get the impression of an edentulous model of the mandible with six implants (Lava COS, 3M, CEREC Ominicam, and Trios 3Shape). Based on the results of this study, the 3M True Definition and Trios scanner demonstrated the highest accuracy. However, the Lava COS was found to be not suitable for taking implant impressions for a cross-arch bridge in the edentulous jaw. This study did not have any conventional group as a control. In our study, Trios scanner had been used which uses the confocal optical imaging technology to generate digital point cloud surfaces.

Regarding the methodology of accuracy measurement, several methods have been employed including the coordinate-measuring machine, traveling microscope, computerized tomography, and optical scanning and digitization. Using digital scanners and the corresponding software represents an efficient method [5]. An industrial metrology 3D scanner (ATOS) with a precision of 4 microns was used in our study. Using the “best-fit algorithm” for superimposing the point cloud is a reliable technique [36].

A limitation of this study is lack of a gauge block for precisely defining the direction of *x-*, *y-*, and *z*-axes though the cylindrical index at the middle of the palate which can play somehow the same role. Moreover, measuring the accuracy of conventional impression and digital impression with two different methods could result in some error related to different precisions of each method. Correlating findings of this in vitro study to clinical situation should be done with caution as there are contributing factors in the oral environment including tissue undercuts, saliva, and limited access during scanning and restricted direction for tray removal.

## 5. Conclusion

With the limitations of this study, the following can be concluded:

1. Digital impression is better than the direct technique in the edentulous arch with straight and tilted implants, and both of them are more accurate than the indirect technique.
2. Type of connection does not have any effect on accuracy when a digital workflow was applied.
3. Precision of implant position also is not affected by the angulation of implants in the digital impression group.

## Figures and Tables

**Table 1:** Definition of groups.

Impression method	Connection type	Group	Number
Digital	Internal	DII	15
	External	DIE	15

Conventional direct	Internal	CDI	15
	External	CDE	15

Conventional indirect	Internal	CII	15
	External	CIE	15

**Table 2:** Mean and SD values of three groups.

Group	Implant angulation	Linear distortion		Angular distortion	
		Mean (mm)	SD	Mean (degree)	SD
DII	Straight	0.188	0.134	0.585	0.724
	Tilted	0.162	0.103	0.364	0.374

DIE	Straight	0.195	0.158	0.587	0.724
	Tilted	0.165	0.134	0.366	0.377

CDI	Straight	0.280	0.142	2.287	1.325
	Tilted	0.389	0.228	4.765	2.203

CDE	Straight	0.711	0.286	1.004	0.453
	Tilted	0.364	0.231	1.098	0.381

CII	Straight	0.885	0.389	4.096	2.726
	Tilted	0.721	0.384	9.371	6.900

CIE	Straight	0.797	0.351	4.851	1.459
	Tilted	0.442	0.226	2.062	0.968

**Table 3:** The effect of the impression technique by comparing inaccuracy values for each group at the implant angulation and connection type.

Impression technique	Internal				External			
	Tilted		Straight		Tilted		Straight	
	Δ*rP* value	Δ*AP* value	Δ*rP* value	Δ*AP* value	Δ*rP* value	Δ*AP* value	Δ*rP* value	Δ*AP* value
Digital versus closed	0.000	0.000	0.000	0.000	0.000	0.000	0.000	0.000
Digital versus open	0.004	0.000	0.35	0.001	0.001	0.000	0.000	0.228
Open versus closed	0.000	0.000	0.000	0.001	0.229	0.000	0.450	0.000

**Table 4:** The effect of the connection type by comparing inaccuracy values for each group at the implant angulation and impression technique.

Impression method	Angulation	Connection type	Group	Δ*rP* value	Δ*AP* value
Digital	Straight	Internal	DII	0.859	0.992
		External	DIE		
	Tilted	Internal	DII	0.916	0.989
		External	DIE		

Conventional direct	Straight	Internal	CDI	0.000^×^	0.000^×^
		External	CDE		
	Tilted	Internal	CDI	0.762	0.000^×^
		External	CDE		

Conventional indirect	Straight	Internal	CII	0.364	0.188
		External	CIE		
	Tilted	Internal	CII	0.001^×^	0.000^×^
		External	CIE		

^×^ *P* value is significant (<0.05).

**Table 5:** The effect of implant angulation by comparing inaccuracy values for each group at the connection type and impression technique.

Impression method		Angulation	Group	Δ*rP* value	∆A *P* value
Digital	Internal	Straight	DII	0.401	0.144
		Tilted			
	External	Straight	DIE	0.433	0.144
		Tilted			

Conventional direct	Internal	Straight	CDI	0.030^×^	0.000^×^
		Tilted			
	External	Straight	CDE	0.000^×^	0.390
		Tilted			

Conventional indirect	Internal	Straight	CII	0.106	0.000^×^
		Tilted			
	External	Straight	CIE	0.000^×^	0.000^×^
		Tilted			

^×^ *P* value is significant (<0.05).

## Data Availability

The data used to support the findings of this study were supplied by Marzieh Alikhasi under license and so cannot be made freely available. Requests for access to these data should be made to Marzieh Alikhasi (m_alikhasi@yahoo.com).
